# Revascularização com Bypass Coronário em Síndromes Coronarianas Agudas sem Supradesnivelamento do Segmento ST: Uma Instantânea de Ensaios e Registros Randomizados

**DOI:** 10.36660/abc.20220248

**Published:** 2022-12-20

**Authors:** Josip Andelo Borovac, Jerko Ferri-Certic, Dino Miric, Jaksa Zanchi, Mislav Lozo, Anteo Bradaric, Konstantin Schwarz, Chun Shing Kwok

**Affiliations:** 1 Cardiovascular Diseases Department University Hospital of Split Split Croácia Cardiovascular Diseases Department, University Hospital of Split, Split – Croácia; 2 Department of Cardiology Dubrovnik General Hospital Dubrovnik Croácia Department of Cardiology, Dubrovnik General Hospital, Dubrovnik – Croácia; 3 Department of Internal Medicine 3 University Hospital St. Polten Karl Landsteiner University of Health Sciences Krems Áustria Department of Internal Medicine 3, University Hospital St. Polten, Karl Landsteiner University of Health Sciences, Krems – Áustria; 4 Royal Stoke University Hospital Stoke-on-Trent Reino Unido Royal Stoke University Hospital, Stoke-on-Trent – Reino Unido

**Keywords:** Angiografia, Infarto do Miocárdio, Antagonistas do Receptor Purinérgico P2Y

Uma recente diretriz da Sociedade Europeia de Cardiologia (ESC) para o manejo de pacientes com síndromes coronarianas agudas (SCA) sem elevação persistente do segmento ST (SCASSST) não recomenda o pré-tratamento de rotina com um antagonista do receptor P_2_Y_12_ em pacientes nos quais a anatomia coronária é indeterminada e o manejo invasivo precoce está planejado (recomendação de classe III, nível de evidência A).^1^ A justificativa para tal recomendação foi baseada principalmente nos resultados obtidos de dois grandes estudos randomizados, ACCOAST^2^ e ISAR-REACT 5,^3^ e a análise da Swedish Coronary Angiography and Angioplasty Registry (SCAAR)^4 , 5^ mostrando que a administração do inibidor de P_2_Y_12_ antes do conhecimento da anatomia coronária em pacientes com SCASSST não melhorou os desfechos isquêmicos e aumentou significativamente o risco de sangramento.

Algumas das preocupações clínicas associadas a pré-tratamento de inibição de plaquetas no SCASSST são baseadas na noção de que tal estratégia pode ser prejudicial em pacientes com outras condições que mimetizam o SCASSST, como dissecção aórtica ou podem estar em risco de sangramento maior ou fatal eventos como hemorragia intracraniana. Da mesma forma, pacientes com SCASSST que precisariam se submeter à cirurgia de revascularização do miocárdio (CRVM) após sua anatomia coronária ser visualizada por angiografia coronária diagnóstica, podem ter risco aumentado de complicações hemorrágicas e atrasos no procedimento devido ao recebimento de pré-tratamento com inibidor de P_2_Y_12_, já que são necessários de 3 a 7 dias para permitir a recuperação da função plaquetária antes da CRVM. Além disso, existem fatores complexos na vida real, como hesitação cirúrgica ou recusa em operar um paciente em um tratamento antiplaquetário duplo atual (DAPT). Deve-se notar também que o sangramento não relacionado à CRVM é uma preocupação relevante para os médicos, como sangramento gastrointestinal ou sangramento de acesso por cateterismo, e essas complicações provavelmente serão mais frequentes com o uso pré-tratamento. Como as últimas diretrizes da ESC afirmam que “ *a estratégia de pré-tratamento pode ser prejudicial para uma proporção relevante* ” desses pacientes, procuramos determinar qual é exatamente a proporção de pacientes com SCASSST que foram encaminhados para cirurgia de revascularização do miocárdio após angiografia e relatamos a prevalência de características de alto risco que possam predispor a um potencial recebimento de revascularização cirúrgica.

Neste trabalho, analisamos deliberadamente dados de ensaios randomizados pivotais que mudaram a prática clínica, citados no documento oficial,^1^ e nos quais a recomendação da diretriz da ESC foi predominantemente baseada. Em seguida, analisamos dados relevantes do mundo real (derivados de registros internacionais ou estudos observacionais que representam a prática clínica em diferentes regiões do mundo). Aqui, relatamos as taxas de revascularização do miocárdio, ICP (intervenção coronária percutânea) e terapia médica ideal (TMO) nesses estudos, bem como características de alto risco que podem estar presentes na população de pacientes com SCASSST, como insuficiência renal, disfunção do VE /insuficiência cardíaca, diabetes mellitus e doença triarterial e/ou lesão do tronco da coronária esquerda (3V/LTCE), conforme relatado e definido pelos autores do estudo. Para isso, calculamos a média ponderada para cada endpoint, ajustada para o tamanho do estudo.

Os estudos randomizados incluíram o estudo ACCOAST^2^ que examinou o pré-tratamento com 30 mg de prasugrel vs. nenhum pré-tratamento em 4.033 pacientes com SCASSST e a subanálise pré-especificada mais recente da coorte de SCASSST5 do estudo de referência ISAR-REACT 5 no qual 2.365 pacientes foram randomizados para receber dose de ataque de 180 mg de ticagrelor antes da angiografia ou dose de ataque de 60 mg de prasugrel administrada no laboratório de cateterismo no momento da angiografia, mas antes da ICP. Além disso, também incluímos dados de estudos randomizados ACUITY^6^ menos contemporâneos que incluíram uma grande coorte de pacientes com SCASSST (N = 13.819), dos quais todos foram submetidos à angiografia em 72 horas. Finalmente, foram incluídos os dados do estudo DUBIUS^7^ recentemente publicado. Este estudo randomizado foi projetado para avaliar os efeitos da administração pré-tratamento vs. downstream do antagonista P_2_Y_12_ entre 1.449 pacientes com SCASSST submetidos à angiografia diagnóstica.

Os dados observacionais e de registro envolvendo pacientes com SCASSST foram derivados de 9 registros em todo o mundo: registro ACTION dos Estados Unidos,^8^ registro ACCEPT do Brasil,^9^ registro multinacional europeu PIRAEUS,^10^ registro ACSIS de Israel,^11^ registro ACS 2 do Canadá,^12^ registro SWEDEHEART da Suécia,^13^ registro CREDO-Kyoto do Japão,^14^ registro ACACIA da Austrália^15^ e um estudo de Desperak et al. relatando dados do grande registro polonês de NSTE-ACS.^16^

**Figura 1:**
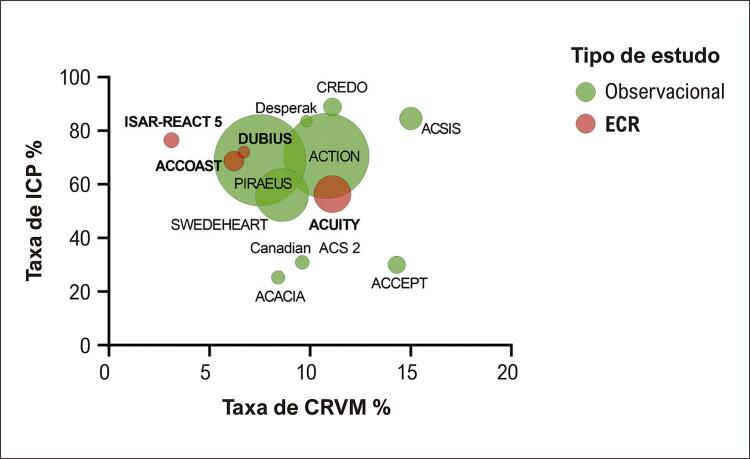
Gráfico de bolhas mostrando a proporção (%) de pacientes com SCASSST tratados com ICP ou CRVM após angiografia diagnóstica em estudos observacionais e ensaios clínicos randomizados (ECR), estratificados por tamanho do estudo. CRVM: cirurgia de revascularização do miocárdio

Quatro estudos randomizados e nove estudos de registro registraram cumulativamente 21.615 e 200.296 pacientes com SCASSST, respectivamente ( Figura Central ). As taxas médias de utilização de ICP em ECRs e estudos de registro foram de 61,9% e 69%, respectivamente, enquanto cerca de 9% dos pacientes com SCASSST foram tratados com cirurgia de revascularização do miocárdio (intervalo de 7,5 a 15% para registros e 3,1 a 11,1% para ECR). As taxas de ambas as intervenções em relação ao tamanho do estudo são mostradas no gráfico de bolhas na Figura 1 .

**Figura Central:**
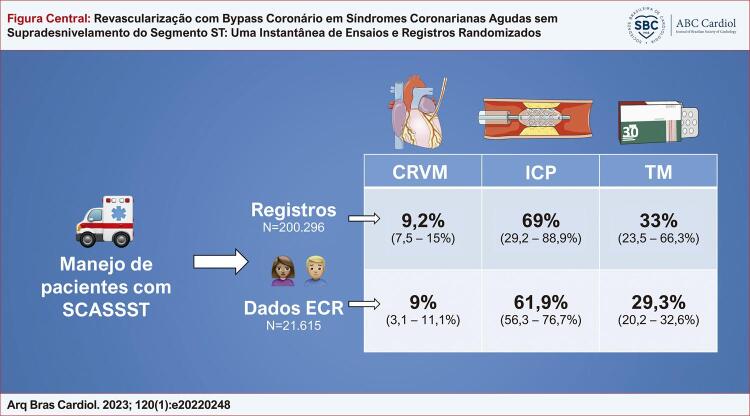
Revascularização com Bypass Coronário em Síndromes Coronarianas Agudas sem Supradesnivelamento do Segmento ST: Uma Instantânea de Ensaios e Registros Randomizados

**Figura 2:**
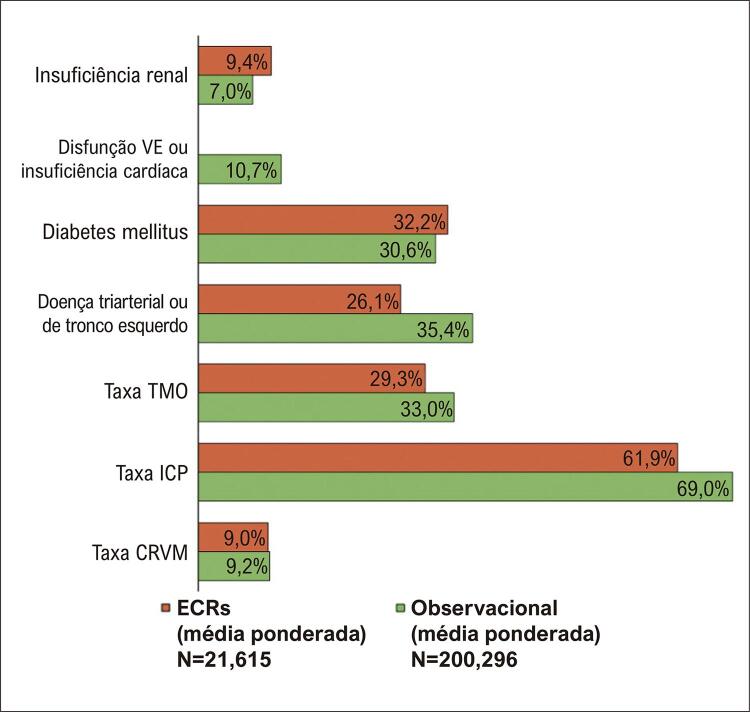
Uma figura resumindo, mostrando a distribuição ponderada das estratégias de manejo (ICP, CRVM, TMO) e características dos pacientes de alto risco, incluindo insuficiência renal, disfunção ventricular esquerda ou insuficiência cardíaca, diabetes mellitus e doença triarterial e/ou de tronco esquerdo entre pacientes com infarto do miocárdio sem supradesnivelamento do segmento. VE: ventrículo esquerdo; CVRM: cirurgia de revascularização do miocárdio; TMO: terapia médica ideal; ICP: intervenção coronária percutânea; ECR: ensaios clínicos randomizados.

Características de pacientes de alto risco, como insuficiência renal, disfunção do VE, diabetes mellitus (insulina e não insulino-dependentes) e 3V/LTCE estavam presentes, em média, em 7%, 10,7%, 30,6% e 35,4% dos casos de SCASSST inscritos em registros ( Figura 2 ). Em contraste, as taxas de insuficiência renal, diabetes mellitus e 3V/LTCE foram de 9,4%, 32,2% e 26,1% entre os pacientes inscritos em estudos randomizados. A prevalência de disfunção VE ou insuficiência cardíaca não estava disponível em estudos randomizados, uma vez que ninguém inscreveu pacientes com insuficiência cardíaca existente ou disfunção sistólica. O material suplementar mostra as características individuais do estudo com mais detalhes.

As características do procedimento dos estudos incluídos parecem concordantes com a prática da vida real, uma vez que uma proporção relevante de pacientes com SCASSST será tratada de forma conservadora, enquanto uma minoria será encaminhada para cirurgia de revascularização do miocárdio. Parece não haver diferença significativa entre ensaios randomizados e registros quanto à taxa de utilização de CRVM; no entanto, deve-se observar uma ampla variação do uso de CRVM entre os estudos. Notavelmente, mais de 10% dos pacientes com SCASSST inscritos nos registros apresentavam disfunção ventricular esquerda ou insuficiência cardíaca, enquanto essa população de pacientes foi excluída de estudos randomizados.

Vários fatores possivelmente impactam as decisões de desencadeamento de ICP vs. CRVM nesta população. Tais fatores podem não ser totalmente dependentes da indicação de intervenção, mas podem ser afetados por caminhos organizacionais no atendimento a pacientes com SCASSST em certos países, disponibilidade de centros de ICP dedicados e cirurgia cardíaca no local. Além disso, fatores paramédicos, como critério do operador, preferências do paciente e políticas de reembolso/seguro em relação aos procedimentos de revascularização realizados no hospital, podem influenciar a escolha da revascularização. No entanto, uma abordagem individualizada e adaptada ao paciente e a tomada de decisão colaborativa envolvendo cardiologistas e cirurgiões cardíacos devem ser incentivadas para alcançar o modo ideal de tratamento.

Uma alta prevalência de características do paciente, como diabetes mellitus, doença multiarterial, função sistólica ruim e insuficiência renal, pode predispor uma proporção significativa de pacientes à revascularização cirúrgica. O panorama geral dos dados também sugere que os pacientes inscritos nos registros tendem a ser mais complexos e têm uma carga de doença maior do que os pacientes inscritos em estudos randomizados. Em relação aos riscos de mortalidade e sangramento, uma recente análise em larga escala do registro SCAAR, incluindo cerca de 65.000 pacientes com SCASSST, todos submetidos a ICP, mostrou que o pré-tratamento com inibidores de P_2_Y_12_ não reduziu os riscos de mortalidade a 30 dias e 1- ano. Ao mesmo tempo, aumentou significativamente o risco de sangramento intra-hospitalar.^4^ Uma análise separada foi então realizada nos dados, incluindo 1.830 pacientes com SCASSST que receberam CRVM – foi demonstrado que as taxas de reoperação por sangramento foram significativamente reduzidas durante o período em que o pré-tratamento com P_2_Y_12_ foi interrompido em comparação com um período em que o pré-tratamento foi praticado rotineiramente. Esses achados de dados observacionais complementam aqueles obtidos em estudos randomizados, como o estudo ISAR-REACT 5, que não mostrou nenhuma vantagem em eficácia se o pré-tratamento for utilizado no cenário de SCASSST.

As limitações de nossa análise são que não é uma revisão sistemática formal – é descritiva e não foram aplicados métodos estatísticos inferenciais. Além disso, detalhes de estudos randomizados, como critérios de inclusão e exclusão e tipos de intervenção e resultados, não foram discutidos em detalhe devido a limitações inerentes ao formato de carta de pesquisa. Da mesma forma, a maioria dos estudos não relatou detalhes importantes sobre anatomia coronariana e indicações para cirurgia de revascularização do miocárdio e se foram realizadas em ambientes eletivos ou de emergência, principalmente porque os estudos não foram focados nesses desfechos. No entanto, os ensaios e registros internacionais mais relevantes e que mudaram a prática foram capturados para gerar uma “instantânea” da prática no cenário SCASSST.

Nossas observações baseadas no registro e dados randomizados corroborariam a recomendação mais recente da ESC de que a suspensão da inibição de P_2_Y_12_ antes da angiografia diagnóstica entre pacientes com SCASSST seria uma abordagem razoável na maioria dos casos. Muitos desses pacientes podem ter uma anatomia coronariana de alto risco e carga de comorbidade, desencadeando assim o encaminhamento para CRVM. A estratégia conservadora P_2_Y_12_ parece particularmente apropriada se esses pacientes recebem cuidados em centros onde o manejo invasivo precoce do SCASSST é acessível e incorporado ao protocolo de rotina. Por outro lado, a incerteza dessa estratégia permanece em cenários clínicos em que os pacientes podem sofrer longos atrasos na angiografia ou transferências para centros habilitados para ICP, como em áreas rurais, ilhas, ou locais sem o suporte de infraestrutura de grandes instituições terciárias. Finalmente, as decisões clínicas sobre o início de pré-tratamento de inibição de P_2_Y_12_ podem ser personalizadas de acordo com as práticas locais/regionais.
