# Supplementary material for: Revascularização com Bypass Coronário em Síndromes Coronarianas Agudas sem Supradesnivelamento do Segmento ST: Uma Instantânea de Ensaios e Registros Randomizados
Source: Arq Bras Cardiol. 2022 Dec 20;120(1):e20220248. [Article in Portuguese] doi: 10.36660/abc.20220248 (PMC9833314; doi:10.36660/abc.20220248)
Supplement: Translation

# Translation: Revascularization with Coronary Artery Bypass Grafting in Non-ST-elevation Acute Coronary Syndromes: A Snapshot of Randomized Trials and Registries

**Authors:** Josip Andelo Borovac, Jerko Ferri-Certic, Dino Miric, Jaksa Zanchi, Mislav Lozo, Anteo Bradaric, Konstantin Schwarz, Chun Shing Kwok

**Affiliations:** 1 Croatia Cardiovascular Diseases Department, University Hospital of Split, Split – Croatia; 2 Croatia Department of Cardiology, Dubrovnik General Hospital, Dubrovnik – Croatia; 3 Austria Department of Internal Medicine 3, University Hospital St. Polten, Karl Landsteiner University of Health Sciences, Krems – Austria; 4 United Kingdom Royal Stoke University Hospital, Stoke-on-Trent – United Kingdom

**Keywords:** Angiography, Myocardial Infarction, Purinergic P2Y Receptor Antagonists

A recent European Society of Cardiology (ESC) guidelines for the management of patients presenting with acute coronary syndromes (ACS) without persistent ST-segment elevation (NSTE-ACS) do not recommend routine pretreatment with a P_2_Y_12_receptor antagonist in patients in which the coronary anatomy is undetermined, and early invasive management is planned (class recommendation III, level of evidence A).^1^The rationale for such recommendation was mainly based on the results obtained from two large randomized trials, ACCOAST^2^and ISAR-REACT 5,^3^and analysis from the Swedish Coronary Angiography and Angioplasty Registry (SCAAR)^4 , 5^showing that administration of P_2_Y_12_inhibitor before the knowledge of coronary anatomy in patients with NSTE-ACS did not improve ischemic outcomes and significantly increased risk of bleeding.

Some of the clinical concerns associated with upstream platelet inhibition in NSTE-ACS are based on the notion that such a strategy might be harmful in patients with other conditions mimicking NSTE-ACS, such as aortic dissection or may be at risk for major or fatal bleeding events such as intracranial bleeding. Likewise, NSTE-ACS patients who would need to undergo coronary artery bypass graft (CABG) surgery after their coronary anatomy is visualized by diagnostic coronary angiography might be at increased risk of bleeding complications and procedural delays due to receipt of P_2_Y_12_inhibitor pretreatment since 3 to 7 days are required to allow for recovery of platelet function prior to CABG. Furthermore, there are complex discretion factors in real-life, such as surgical hesitancy or decline to operate on a patient on a current dual antiplatelet treatment (DAPT). It should also be noted that non-CABG-related bleeding is a relevant concern for clinicians, such as gastrointestinal bleeding or catheterization access bleeding, and these complications are likely to be more frequent with pretreatment use. Since the latest ESC guidelines state that *“pretreatment strategy might be harmful to a relevant proportion “* of such patients, we sought to determine what exactly is the proportion of patients with NSTE-ACS that were referred to CABG surgery following angiography and we report on the prevalence of high-risk features that might predispose them to a potential receipt of surgical revascularization.

In this work, we deliberately analyzed data from pivotal randomized trials that changed clinical practice, cited in the official document,^1^and on which the ESC guideline recommendation was dominantly based. We then analyzed relevant real-world data (derived from international registries or observational studies representing clinical practice in different regions of the world). Herein, we report on the rates of CABG, PCI, and optimal medical therapy (OMT) across these studies, as well as high-risk characteristics that might be present in the population of patients with NSTE-ACS such as renal failure, LV dysfunction/heart failure, diabetes mellitus and three-vessel and/or left main coronary disease (3V/LMD), as reported and defined by the study authors. For this purpose, we calculated the weighted mean for each endpoint, adjusted for study size.

Randomized studies included the ACCOAST^2^trial that examined the 30 mg prasugrel pretreatment *vs.* no pretreatment in 4033 patients with NSTE-ACS and the most recent prespecified subanalysis of NSTE-ACS cohort^5^of landmark ISAR-REACT 5 trial in which 2365 patients were randomized to receive 180 mg loading dose of ticagrelor before angiography or 60 mg loading dose of prasugrel administered in the cath lab at the time of angiography but before PCI. Moreover, we also included data from less contemporary ACUITY^6^randomized trials that enrolled a large cohort of NSTE-ACS patients (N=13,819), of which all underwent angiography within 72 hours. Finally, data from the recently published DUBIUS^7^trial were included. This randomized trial was designed to evaluate the effects of upstream (pretreatment) *vs* . downstream administration of P_2_Y_12_antagonist among 1449 patients with NSTE-ACS that underwent diagnostic angiography.

Observational and registry data enrolling patients with NSTE-ACS were derived from 9 registries worldwide: ACTION registry from United States,^8^ACCEPT registry from the Brazil,^9^PIRAEUS multinational European registry,^10^ACSIS registry from Israel,^11^ACS 2 registry from Canada,^12^SWEDEHEART registry from Sweden,^13^CREDO-Kyoto registry from Japan,^14^ACACIA registry from Australia,^15^and a study by Desperak and colleagues reporting data from the large Polish registry of NSTE-ACS.^16^

Four randomized trials and nine registry studies cumulatively enrolled 21,615 and 200,296 patients with NSTE-ACS, respectively ( Central Figure ). The average rates of PCI utilization in RCTs (randomized controlled trials) and registry studies were 61.9% and 69%, respectively, while about 9% of patients with NSTE-ACS were treated with CABG surgery (range of 7.5 to 15% for registries and 3.1 to 11.1% for RCTs). Rates of both interventions concerning study size are shown in the bubble plot in Figure 1 .

**Central Illustration:** Revascularization with Coronary Artery Bypass Grafting in Non-STelevation Acute Coronary Syndromes: A Snapshot of Randomized Trials and Registries

**Figure 1:** A bubble plot showing the proportion (%) of patients with NSTE-ACS treated with PCI or CABG after diagnostic angiography in observational studies and randomized controlled trials (RCTs), stratified by study size. CABG: coronary artery bypass graft.

High-risk patient characteristics such as renal failure, LV dysfunction, diabetes mellitus (both insulin and non-insulin-dependent), and 3V/LMD were, on average, present in 7%, 10.7%, 30.6%, and 35.4% of NSTE-ACS cases enrolled in registries ( Figure 2 ). In contrast, rates of renal failure, diabetes mellitus, and 3V/LMD were 9.4%, 32.2%, and 26.1% among patients enrolled in randomized trials. Prevalence of LV dysfunction or heart failure was unavailable in randomized studies since none enrolled patients with existing heart failure or over systolic dysfunction. The Supplemental material shows individual study characteristics in more detail.

**Figure 2:** A summarizing figure showing the weighted distribution of management strategies (PCI, CABG, OMT) and high-risk patient characteristics, including renal failure, left ventricular dysfunction or heart failure, diabetes mellitus and three-vessel and/or left main disease among patients with NSTE-ACS. CABG: coronary artery bypass graft; NSTE-ACS: non-ST-elevation myocardial infarction; OMT: optimal medical therapy; PCI: percutaneous coronary intervention.

Procedural characteristics of included studies seem concordant with real-life practice since a relevant proportion of NSTE-ACS patients will be treated conservatively while a minority will be referred to CABG surgery. There seems to be no significant difference between randomized trials and registries concerning the rate of CABG utilization; however, a wide variation of CABG use across studies should be noted. Notably, more than 10% of patients with NSTE-ACS enrolled in registries had left ventricular dysfunction or heart failure, while this patient population was excluded from randomized trials.

Various factors possibly impact decisions triggering PCI vs. CABG in this population. Such factors might not be entirely dependent on the interventional indication but could be affected by organizational pathways in care for NSTE-ACS patients in certain countries, availability of dedicated PCI centers, and on-site cardiac surgery. Also, paramedical factors such as an operator’s discretion, patient preferences, and reimbursement/insurance policies regarding revascularization procedures performed in the hospital might influence the choice of revascularization. However, an individualized patient-tailored approach and collaborative decision-making involving cardiologists and cardiac surgeons should be encouraged to reach the optimal mode of treatment.

A high prevalence of patient characteristics such as diabetes mellitus, multivessel disease, poor systolic function, and renal failure might predispose a significant proportion of patients to surgical revascularization. The general snapshot of the data also suggests that patients enrolled in registries tend to be more complex and have a higher disease burden than patients enrolled in randomized trials. Concerning risks of mortality and bleeding, a recent large-scale analysis of the SCAAR registry, including nearly 65,000 patients with NSTE-ACS, of whom all underwent PCI, showed that pretreatment with P_2_Y_12_inhibitors did not reduce risks of 30-day and 1-year mortality. At the same time, it significantly increased the risk of in-hospital bleeding.^4^A separate analysis was then performed on the data, including 1830 patients with NSTE-ACS that received CABG – it was demonstrated that rates of reoperation due to bleeding were significantly reduced during the period in which P_2_Y_12_pretreatment was halted compared to a period in which pretreatment was routinely practiced. These findings from observational data complement those obtained from randomized trials, such as ISAR-REACT 5 trial, which showed no advantage in efficacy if pretreatment is utilized in the NSTE-ACS setting.

The limitations of our analysis are that it is not a formal systematic review – it is descriptive, and no inferential statistical methods were applied. Furthermore, details of randomized trials, such as inclusion and exclusion criteria and types of intervention and outcomes, were not discussed in detail due to limitations inherent to a research letter format. Similarly, most studies did not report important details on coronary anatomy and indications for CABG surgery and whether they were performed in elective or emergent settings, mostly because studies were not focused on these endpoints. However, the most relevant and practice-changing trials and international registries were captured to generate a “snapshot “of practice in the NSTE-ACS setting.

Our observations based on the registry and randomized data would corroborate the latest ESC’s guideline-directed recommendation that withholding P_2_Y_12_inhibition before diagnostic angiography among patients with NSTE-ACS would be a reasonable approach in most instances. Many of these patients may have a high-risk coronary anatomy and comorbidity burden, thus triggering CABG referral. Conservative P_2_Y_12_strategy particularly seems appropriate if these patients receive care at centers where early invasive management of NSTE-ACS is accessible and incorporated into the routine protocol. On the other hand, the uncertainty of this strategy remains in clinical scenarios in which patients might experience long delays to angiography or transfers to PCI-capable centers, such as in rural areas, islands, or locations without the infrastructural support of large tertiary institutions. Finally, clinical decisions on initiating upstream P_2_Y_12_inhibition might be customized according to local/regional practices.
